# Glioblastoma Metabolomics—In Vitro Studies

**DOI:** 10.3390/metabo11050315

**Published:** 2021-05-13

**Authors:** Karol Jaroch, Paulina Modrakowska, Barbara Bojko

**Affiliations:** Department of Pharmacodynamics and Molecular Pharmacology, Faculty of Pharmacy, Collegium Medicum in Bydgoszcz, Nicolaus Copernicus University in Toruń, dr A. Jurasza 2 Street, 85-089 Bydgoszcz, Poland; karol.jaroch@cm.umk.pl (K.J.); p.modrakowska@cm.umk.pl (P.M.)

**Keywords:** glioblastoma multiforme, in vitro metabolomics, phamacometabolomics

## Abstract

In 2016, the WHO introduced new guidelines for the diagnosis of brain gliomas based on new genomic markers. The addition of these new markers to the pre-existing diagnostic methods provided a new level of precision for the diagnosis of glioma and the prediction of treatment effectiveness. Yet, despite this new classification tool, glioblastoma (GBM), a grade IV glioma, continues to have one of the highest mortality rates among central nervous system tumors. Metabolomics is a particularly promising tool for the analysis of GBM tumors and potential methods of treating them, as it is the only “omics” approach that is capable of providing a metabolic signature of a tumor’s phenotype. With careful experimental design, cell cultures can be a useful matrix in GBM metabolomics, as they ensure stable conditions and, under proper conditions, are capable of capturing different tumor phenotypes. This paper reviews in vitro metabolomic profiling studies of high-grade gliomas, with a particular focus on sample-preparation techniques, crucial metabolites identified, cell culture conditions, in vitro-in vivo extrapolation, and pharmacometabolomics. Ultimately, this review aims to elucidate potential future directions for in vitro GBM metabolomics.

## 1. Introduction

Glioblastoma (GBM) is one of the most aggressive and difficult-to-treat central nervous system (CNS) brain tumors. Since 2007, the World Health Organization (WHO) has classified gliomas based on their cell type and aggressiveness, with Class I consisting of benign tumors, and Class IV comprising the most aggressive types of tumors. GBM is a Class IV brain tumor [1]. While this classification system allows clinicians to determine appropriate treatments and prognoses, years of studies have indicated that this approach should be supplemented with genetic testing, as it lacks adequate specificity on its own. As a result, in 2016 the WHO introduced a novel CNS grading system that provided a level of precision surpassing all known CNS diagnostic and classification methods. This novel grading system incorporated new genetic markers—for example, IDH1/IDH2, *O^6^* -mtehylguanine DNA methyltransferase (MGMT), and epidermal growth factor receptors (EGFR)—thereby allowing clinicians to differentiate tumors not only by their cell type and aggressiveness, as was possible with pre-existing methods, but also by the genetic phenotype of the neoplastic cells, thus providing better correlation with the tumor prognosis [2]. Despite this new, improved diagnostic system, GBM continues to be the most lethal primary malignant CNS tumor. Indeed, in the USA, patients diagnosed with GBM have an average life-expectancy of eight months, with only 7.2% surviving beyond five years of diagnosis [3].

The treatment of GBM remains a challenge, as newly proposed drugs must meet specific requirements, such as being able to cross the blood-brain barrier (BBB) and efficiently infiltrating the tumor. GBM tumors are known for their complex structure, which is the result of a demanding growth environment. Other features of GBM tumors that make them so challenging to treat include high proliferation indices, angiogenesis, and pseudopalisading necrosis [4]. Intratumoral hypoxia is caused by rapid cell proliferation and vascular collapse, and it induces the expression of hypoxia-inducible factor-1 (HIF-1), which is responsible for regulating many key processes involved in tumor progression and invasion. Among these processes, metabolic reprogramming appears to be critical in understanding the resistance of GBM tumors to chemotherapy and radiation therapy [5]. The most commonly used method of treating GBM is tumor resection followed by radiation therapy and/or chemotherapy with temozolomide (TMZ) [6], an alkylating agent that targets cells undergoing intense proliferation. TMZ works by inducing DNA methylation, which in turn arrests the cell cycle and, consequently, induces apoptosis, autophagy and senescence [7]. Since the methylation of the *O^6^* position of guanine caused by TMZ can only be repaired by the enzyme, MGMT [8], tumors expressing MGMT may exhibit a natural resistance to TMZ. However, resistance to TMZ can still develop over time, even in tumors that responded positively to treatment with it. Studies examining the role of hypoxia in TMZ resistance have found that, while hypoxia mediates some important processes that facilitate TMZ resistance in GBMs, the tumors can be resensitized via hyperoxia [9,10,11]. Similarly, anti-angiogenesis-based therapies such as targeted therapy using the vascular endothelial growth factor (VEGF) inhibitor, bevacizumab are also susceptible to the same problem of resistance due to hypoxia. As with TMZ, bevacizumab resistance has also been linked to hypoxia [12,13]. Moreover, GBM tumors are difficult to treat due to their heterogeneous nature. In particular, their concentration of glioma stem-like cells (GSCs) can pose a distinct challenge, as these cells possess properties that allow them to change their cellular phenotypes in response to existing microenvironment conditions. This plasticity has also been linked to hypoxia [14,15]. The key role played by hypoxia in regulating the microenvironments of many different types of tumors has led researchers to focus greater amounts of attention on the potential of therapies targeting hypoxic regions [16].

The metabolomic reprogramming of cancer cells is a well-known phenomenon. The stressful environment created by hypoxia generally impairs oxidative phosphorylation and TCA cycle activity in the intensely proliferating tumor cells and enhances glycolysis and lactic acid production. This phenomenon, also known as the Warburg effect, is indirectly strengthened by HIF-1 expression in hypoxic environments. However, it remains unclear how exactly hypoxia influences the metabolomic reprogramming of tumor cells. As such, the development of models that more accurately represent tumor microenvironment metabolomics is required [17,18,19].

Metabolomics, along with genomics, transcriptomics, and proteomics, comprise the group of sciences known as “Omics.” Metabolomics focuses on the analysis of small molecules (<1.5 kDa) produced as a result of metabolism [20]. It is possible to obtain a relatively full picture of the state of a given cell or tissue by analyzing its endogenous and exogenous metabolites [21]. The great advantage of metabolomics is that the metabolome accurately mirrors the phenotype and influence of factors external to the analyzed cell, which cannot be captured as precisely with genomics or proteomics [22].

Recently, in vitro studies using both established GBM cell lines and primary GBM cells have been gaining in popularity due to rapid developments in 3D in vitro culture techniques. One reason for this surge in popularity is that 3D culture systems provide a more accurate microenvironment, as they capture important cell-matrix and cell-cell interactions that are absent from cells cultured as a two-dimensional monolayer (2D) [23,24]. However, there are many challenges that must be overcome in order to efficiently conduct metabolomic research using in vitro cell cultures, both 2D and 3D. For example, metabolomics requires careful experimental design with regards to cell culture normalization, cell disruption, metabolism quenching, and metabolome extraction [25]. 2D cell culture is a well-known model for in vitro studies that is easier to normalize, opposed to 3D cell cultures, where each cell spheroid can have different cell number, size, and shape. Standard monolayer culture is also easy to conduct, as protocols for culturing and testing 2D cell cultures were well established through the years. In turn, 3D cell culture reflects in vivo tumor complexity better, yet it is a relatively new culture method and standard culturing and testing protocols are yet to be established. Nevertheless, with appropriate experimental design, metabolomics of GBM cell cultures can deliver information about alternate metabolic pathways, potential biomarkers, and with proper in vitro-in vivo extrapolation (IVIVE), drug development and repurposing.

This review provides an overview of the major sample-preparation methods for metabolomics analysis, and analyzes promising metabolomics studies with GBM cell lines within the context of the potential biomarkers, therapeutic targets, and IVIVE.

## 2. Sample Preparation for In Vitro Studies

Investigations of the metabolomes of various GBM cell lines consist of two parts: extracellular and intracellular. The extracellular investigation is performed using a cell-culture medium that is simply pulled after cell growth, followed by an optional centrifugation step and the addition of an organic solvent for LC-MS and GC analysis (e.g., methanol, acetonitrile) [26,27,28]. An additional derivatization step is required for GC analysis [27,29], while medium filtration with either deuterated water [30], deuterated water with sodium 3-trimethylsilyl [2,2,3,3-2H4] propionate (TMSP), sodium 3-(trimethylsilyl)propionate-2,2,3,3-d4 (TSP), or sodium (2,2-dimethyl-2-silapentane-5-sulfonate) (DSS) is required for nuclear magnetic resonance (NMR) analysis [31]. The extracts are subjected to ultracentrifugation prior to LC and GC analysis in order to remove proteins and debris (e.g., from serum used in medium or cell debris). In one case, extracellular amino acid profiling was performed via protein precipitation with sulfosalicylic acid, followed by labelling with aTRAQ^TM^ agent [32].

The first step in most documented intracellular analysis protocols entails washing the sample in cold PBS solution in order to quench the metabolism of cells, which prevents alterations to metabolomics patterns from further manipulation. After this initial metabolism-quenching step one of two major approaches can be employed: examining cell detachment, or directly applying cold organic solvent to the surface of the growing cells. Cell detachment is assessed via trypsinization or manual cell scraping, followed by the addition of a solvent. These two steps are sometimes combined by adding the organic solvent directly onto the cell culture plate/Petri dish, followed by cell scraping. Next, the sample is transferred into tubes and vortexed/shaken, followed by ultracentrifugation in order to remove any debris. After ultracentrifugation, the samples are evaporated and either (1) reconstituted with a solvent that is compatible with liquid chromatography, (2) derivatized and injected on gas chromatography, or (3) reconstituted with deuterated water spiked with TSP [33,34,35], TMSP [31,36,37], DSS [38], and propionic-2,2,3,3,-d4 acid [38] or TMS [39] for nuclear magnetic resonance analysis. Aside from the above-described simple liquid-liquid extraction approach, researchers have also employed a dual-phase extraction approach. Briefly, this protocol entails the sequential addition of methanol, chloroform, and water (adding order varied) to a final ratio of 1:1:1 v/v/v, followed by sample mixing and centrifugation to separate the upper phase, which contains water-soluble polar metabolites, from the lower chloroform phase, which contains non-polar/lipid compounds. After separation, one or both phases are transferred into separate vials, evaporated, and reconstituted. The methanol:water phase can be further cleaned using divalent ions from Chelex-100 resin [40]. Another unique approach was developed by Izquierdo-Garcia et al. [33], wherein U87 and Normal Human Astrocytes (NHA) cells were incubated in a medium containing 1-13C-glucose or L 3-13C-glutamine (Gln) in order to allow these isotopes to be incorporated into low-molecular-mass compounds, which were further determined via 13C-MRS. In addition, Izquierdo-Garcia et al. [32] also used 2-13C-pyruvic acid for their hyperpolarized 13C-MRS experiments. They performed their MRS experiments using a perfusion system, which enabled the medium to circulate from the cells immobilized on the bead and into a 10-mm MR tube [33,41]. Summarizing, sample preparation among described articles is not sophisticated as the extraction is driven by the partitioning of compounds from sample into an organic solvent. Next, a clean-up is performed, in most cases by centrifugation, followed by manipulation needed for particular instrumental platform, e.g., evaporation and resuspension in deuterated water for NMR or derivatization for GC, etc. Despite the simplicity, a high number of compounds were found and described by authors. An updated list of the sample preparation methods for an in vitro extra- and intracellular metabolome are described in Table 1 and Table 2. 

## 3. Metabolomics of GBM In Vitro

Many recent studies on the development of tumor malignancy and resistance to treatment have focused on the metabolic reprogramming of cancer cells. Investigations into the metabolomic phenotype of various tumors, including brain tumors, have revealed interesting correlations between a tumor’s mutations, metabolic footprint, and microenvironment [60,61]. Given these correlations, metabolomics and lipidomics may be effective tools in drug development and brain tumor diagnostics, grading, and prognosis [61,62]. Prior studies have successfully detected numerous metabolic alterations, particularly in relation to the metabolism of fatty acids and amino acids, such as Gln, choline (Cho), and cysteine (Cys) [63,64,65,66]. However, these findings represent only a small fraction of the work that has been done in GBM metabolomics and lipidomics—a body of work that is constantly growing, as researchers continue to work to identify important metabolites in GBM development. Generally, studies examining the metabolic reprogramming of cancer have utilized matrices such as blood and serum, urine, tissue samples, and established cell lines and primary cells [60,61]. While all of these matrices have been successfully employed, in vitro studies using both established cell lines and primary cells ensure replicable and strictly controlled conditions between each replicate sample. Furthermore, the analysis of culture media and disintegrated cells, along with careful sample preparation, can provide useful information about both the endo- and exo-metabolome. However, cells growing in vitro as a monolayer do not adequately recreate the tumor microenvironment. As such, researchers have increasingly been exploring the use of three-dimensional (3D) in vitro culture models, as they reflect the actual tumor phenotype more adequately than standard 2D cell cultures [67]. For these reasons, in vitro cell cultures remain of great interest in explorations of metabolic reprogramming in GBM tumors. For the sake of clarity, from now on when discussing metabolic studies on in vitro cell cultures it will refer to the 2D culture model, as it is still considered the standard in in vitro studies, unless specified otherwise.

Metabolic alterations in cancer cells have long been explored for their usefulness in profiling of the phenotypes of many different types of tumors [68]. Prior to the development of the WHO glioma tumor classification method, researchers obtained information about different patterns in the metabolic pathways between normal and malignant cells through simple in vitro studies using established GBM cell lines (U87) and human mesenchymal stem cell lines (hMSC) [46]. In their work on intracellular metabolomes, Juerchott et al. observed alterations in the TCA cycle, with amplified concentrations of fumarate and succinate, and lower concentrations of citrate [46]. In addition, Juerchott et al. also observed that some glycolysis metabolites, such as glucose-6-phosphate (G6P), were upregulated. Many of the metabolites detected in their study would appear in later studies, not only for grading GBMs and determining prognosis, but also for determining drug treatment efficiency.

Findings have also revealed good correlation between mutations found in GBM, e.g., PDGFRA, IDH1, EGFR, and NF1—and the tumor’s metabolic fingerprint. Cuperlovic-Culf et al. conducted metabolic profiling on nine established GBM cell lines and categorized them into four subtypes based on the alterations to their metabolites [30]. Their findings proved that it is necessary to monitor alterations in metabolic pathways instead of focusing on DNA mutations alone. For instance, alterations to Cho—which is known to be present in cancer cells at different concentrations than in normal cells—and its derivatives (phosphocholine (PC) and glycerophosphocholine (GPC)) were only observed in the first group of cell lines [30]. The cells in this group had a genetic profile of PDGFRA+ and EGFR-, as well as significantly higher concentrations of Cho, PC, and GPC. Izquierdo-Garcia et al.’s examination of IDH1-mutated U87 GBM cells found decreased concentrations of PC and increased concentrations of GPC [33]. Since IDH1 mutations are generally more common in low-grade gliomas, the general ratio of PC to GPC could serve as a prediction factor, such that elevated levels of PC and decreased levels of GPC would indicate high-level gliomas, such as GBM [69,70]. Moreover, a low lipids-to-GPC ratio was found to connect patient-derived cell lines and neural progenitor cells; as such, this ratio can be used to characterize the neural phenotype of the tumor, and thus discern a better prognosis [37]. Another study revealed a correlation between the upregulation of GPCs and Cho and the differentiated state of the cells. This finding implies that impaired glycophospholipids metabolism is correlated with the tumor self-renewal and, thus, a worse prognosis [42]. Furthermore, a comparison of PC and GPC levels in pediatric GBM tumors and tumor-derived cells showed a decrease in the levels of both metabolites in both late passage cell lines and the tumor at relapse, indicating that both the tumor and derived cells had transitioned from stem-like cells into differentiated cells [52]. Nonetheless, it remains an open question whether a low PC-to-GPC ratio is a clear indicator of low malignancy grade in gliomas, with research still ongoing to determine the efficacy of these two metabolic markers. However, the ratio of total Cho to total creatine is indeed an indicator of the worse prognosis [38,71].

Inositol and myo-inositol are two additional metabolites that could potentially be useful in GBM diagnostics and prognostics, as they are known to play roles in osmoregulation and phosphatidylinositol lipids synthesis [72]. In a study conducted by Cuperlovic-Culf et al. a correlation was observed between the upregulation of myo-inositol and the PDGFRA+ and EGFR- genotypes in one of these subtypes [30]. Conversely, findings have shown that IDH mutant cells have decreased myo-inositol levels compared to an IDH wild-type cell line [33]. Kahlert et al. reported a high myo-inositol-to-glycine ratio for a U87 cell line grown in neutrospheres, which could be a marker for GBM [38]. Moreover, since myo-inositol plays a role in the metabolism of glycerophospholipids, its high concentration could be explained by the self-renewing properties of GBM tumors [42,73]. On the basis of the research discussed, it can be concluded that elevated levels of myo-inositol could be markers indicating high grade glioma.

Gln, glutamate (Glu), and γ-amniobutyric acid (GABA) each play an extremely important role in brain development. Changes in the metabolism of Gln can cause disturbances in Glu, GABA, and aspartate (Asp), as it is the precursor of these neurotransmitters [74]. Furthermore, Gln can be converted into α-ketoglutarate (α-KG), which subsequently takes part in the TCA cycle [75]. Tardito et al. highlighted GBM’s dependency on Gln. Their findings indicated that synthesized Gln can be used to synthesize AMP [26]. In their study, Cuperlovic-Culf et al. determined that differences in the upregulation of Gln, Glu, Asp, and citrate were dependent on the subtype of the studied cell lines [30]. They found that the levels of these metabolites in each subtype correlated with the expression of genes for some transporters such as SLC38A1, SLC7A8, and SLC1A. Specifically, they found that the overexpression of certain cellular or mitochondrial transporters influenced the levels of these metabolites. In turn, decreases in Gln were associated with IDH1mut status [33], and enforced glutaminolysis was connected to the ASS negative cell lines [29] and the accelerated growth rate of Gln-dependent GBM cells [32]. Glutaminolysis tends to be also overexpressed in relapse tumors and cells grown in nerurospheres [52]. A study on IDH wild-type primary GBM cell cultures yielded similar results, with two clear subtypes emerging: one with increased Gln uptake, and another with low Gln uptake. The findings showed that this high Gln dependency was correlated with a mesenchymal-type tumor and the worst prognosis [32]. In another study, Guidoni et al. compared patient-derived cells to GBM cell line T98G and neural stem/progenitor cells. They observed that the levels of GABA in one of the patient-derived cell lines increased while Glu simultaneously decreased, which could be used to determine the neuronal phenotype, as GABA synthesis mainly takes place in the neurons [37,74]. Moreover, the presence of neuronal metabolic markers is correlated with better prognoses [37].

Glutathione (GSH) is a tripeptide that is composed of Glu, Cys, and glycine (Gly). GSH can take on two forms, namely reduced GSH and oxidized GSSG, which allows it to play an important role in redox regulation and protecting cells from ROS [76]. The up-regulation of GSH has been associated with groups of cell lines from WHO grade IV gliomas, which connects it to the malignant transformation of the tumor [34]. A comparison of stem-like U87MG cells to U87 malignant glioma cells and stem-like cells after induced differentiation revealed a drop in GSSG levels and a high GSH-to-GSSG ratio. Therefore, low levels of ROS metabolites could be associated with worse prognoses, while increased levels of these metabolites could induce the differentiation of stem-like cells in tumors [42]. Similarly, decrease in GSH has been associated with the IDH1mut genotype of the U87 cell line [33]. Low GSH levels have also been observed in cells grown in neurospheres, which show more astrocyte/glioma-like metabolism. This finding indicates that decreased GSH is connected to hypoxia, and thus a worse prognosis, as was confirmed by the study’s patient results [37]. However, one needs to remember that GSH easily undergoes auto-oxidation during the sample preparation step, what makes it easy to get false results [77]. To the best of our knowledge, there is no GBM study which highlights this problem, the solutions proposed based on other cell cultures, i.e., adding N-ethylmaleimid and acetonitrile directly after removing the culture medium form the culture flask, can be considered in the in vitro GBM studies [78].

Studies performed on glioma cell models have successfully connected the widely known glioma marker, 2-hydroxyglutarate (2-HG) with the IDH1 mutation, as IDH-mutated cells gained a new, unique ability to convert α-KG into 2-HG, that IDH-wildtype glioma cells do not possess [30]. Live cell monitoring with 13C-MRS revealed elevated concentrations of 2-HG in the IDH1mut cells, along with a simultaneous drop in Glu concentrations [33]. This correlation was further explored in another study, where it was confirmed that 2-HG requires glucose in addition to Glu [45]. 2-HG is a good oncotarget for use in differentiating low-grade gliomas from GBMs, with Gln and glucose deprivation serving as useful therapeutic targets for such analyses.

**Figure 1:**
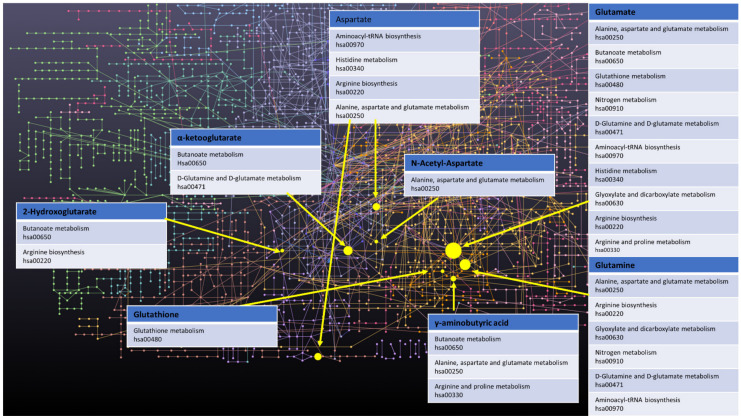
Network of GBM related oncometabolites. Network generated with the MetaboAnalyst 5.0 online [79], pathways names and codes from Kyoto Encyclopedia of Genes and Genomes database [80].

Finally, a few other metabolites and altered pathways, such as N-acetyl aspartate (NAA), have been suggested as important for GBM metabolomic diagnostics, prognosis, and drug testing [37,52]. The full scope of important in vitro GBM metabolites analysed is presented in Table 1. Moreover, key metabolites that have been discussed in this paragraph, i.e., α-KG, 2-HG, Gln, Glu, GABA, GSH, and Asp, were analysed with the MetaboAnalyst 5.0 online. The most prevalent metabolic pathways are shown in the Figure 1, where Glu and Gln appear most often, suggesting them as metabolites important for the disease in question, while arginine metabolism and biosynthesis, Asp, D-Gln, and D-Glu metabolism are the most dominant pathways. To summarize metabolites such as Co, PC, GPC, myo-inositol, Gln, Glu, GABA, Asp, α-KG, GSH and 2-HG could be all used for GBM grading. Elevated myo-inositol, high Gln and Glu dependency and decrease in GSH could all indicate high grade glioma, while high 2-HG concentration could be associated with IDH1 mutation and therefore better prognosis. However, the most optimal solution would be to create a panel of key metabolites and analyze not only changes in levels of those, but also ratios between them.

## 4. Importance of GBM Microenvironment Reconstruction for In Vitro Metabolomics

GBM is a tumor that is known to have a highly complicated microenvironment, largely due to its heterogeneous nature, intratumor hypoxia, and angiogenesis [14,15]. Therefore, to carry out metabolomic in vitro studies that will translate to an in vivo environment, it is extremely important to consider culture conditions and cell source in metabolomic testing. For patient-derived GBM cells, special culture conditions, such as the use of an FBS-free culture medium supplemented with growth factors, as well as the use of 3D culturing in neurospheres, are recommended in order to acquire cells that actually feature all tumor characteristics [24,81,82]. 3D culture was more favorable for stem-like cells (CD133+). Furthermore, the cells in the 3D culture were also characterized by higher tCho-to-tCre, Gly-to-myo (myo-inositol), and Gly-to-tCho ratios, which are all indicators of high-grade gliomas [38]. In a similar, more recent study, Pexito et al. extended this investigation. They observed significant alterations in arginine metabolism in the cell lines that were cultured in the neurospheres [59]. Moreover, a comparison of patient-derived cells cultured in neutrospheres actually reflected the metabolic fingerprint of relapsed tumors [52]. Notably, neurospheres were used to culture glioma stem-like cells in many of the studies discussed in the current review (Table 1) [27,32,35,37,45,52,54].

Hypoxia is a common phenomenon in cancers, but it remains difficult to replicate hypoxic environments in vitro. Spheroid formation is one method that can be used to create low-oxygen conditions in cultures, as the core of the spheres is naturally hypoxic. However, this approach does not ensure the replicable conditions that are required in certain types of studies. These conditions can be achieved by lowering the O_2_ content in the culture environment using equipment such as a CO_2_ incubator. The profiling of U87MG cells grown in both hypoxic and normoxic environments revealed that hypoxia induces the non-glycolytic metabolism of glucose, which suggests that glycoproteins and glycolipids can be used as markers for hypoxia in GBM tumors. Moreover, the authors of the study further observed alterations to the TCA cycle, 2-HG accumulation, the altered metabolism of lipids, and increased catabolism of amino acids in hypoxic GBM cells [57]. A separate analysis of primary cell culture in hypoxic conditions revealed that oxygen deprivation induces changes in the α-KG-to-succinate ratio, as well as the Gly content [56]. Finally, Blandin et al. showed that cells cultured in hypoxic conditions more closely resembled the actual metabolomic profile of a tumor [52]. Therefore, in order to pursue a truly accurate metabolomic analysis of GBM in vitro, it should be taken into account that standard culture conditions established over the years, e.g., 2D cell culture, culture medium supplemented with FBS, and normoxic conditions, do not accurately reflect the complexity of the tumor. When planning the experiment, it is advisable to conduct simultaneous experiment with the use of 3D cell culture, FBS-free medium and under hypoxic conditions.

## 5. In Vitro-In Vivo Extrapolation of Oncometabolites

To date, several low-molecular-weight compounds have been identified as possible biomarkers of GBM. In particular, the dysregulation of the oncometabolites, 2-HG [83,84,85], NAA [86], Glu [64], and α-KG) [64] has been shown to be connected to the altered enzymatic pathways that occur within cancerous cells. Thus, these low-molecular-mass compounds are potential targets for in vitro-in vivo extrapolation. All of the above-mentioned compounds were identified through a literature search. As mentioned above, 2-HG and Glu were found via live cell monitoring using 13C-MRS, wherein cell culture medium was supplemented with 3-13C-glutamine. This enabled the determination of 13C-Glu and 13C-2-HG in U87IDHmut, and the determination of 13C-Glu only in U87IDHwt cells [45]. Consequently, in terms of IVIVE, Glu and 2-HG can serve not only as GBM biomarkers, but also as markers of *IDH1* mutation, which plays key role in chemotherapy treatment optimization. In another study, researchers determined 2-HG through the extraction of intracellular components, followed by NMR analysis [33]. NAA and Glu were successfully found via NMR as the effect of intracellular metabolome investigation within cell cultures established from tissue of pediatric origin derived by NMR cell culture model [52], primary glioblastoma stem-like cells (GSC) [37] and GL261 cell line. Guidoni et al., observed that NAA was not present in the GBM T98G cell line, which suggests that primary GSC is closer to the in vivo state [37]. Glu was also identified in pediatric low-grade glioma using an LC-MS approach, wherein everolimus treatment resulted in glutaminase inhibition, which in turn led to reduced Glu levels [49], as well as result of extracellular metabolome study of U87-MG cell line [32]. Researchers have also utilized LC-MS/MS to analyse and compare Glu secretion and consumption in a medium-based extracellular metabolome and a cell-lysate-based intracellular metabolome [26]. The dual-phase extraction of intracellular components of U87 followed by GC-MS also revealed presence of Glu, which was observed at higher levels compared to normal hMSCs within the U87 cell line [46]. TMZ treatment caused difference in Glu levels between drug resistant and drug sensitive primary GBM cells with increased Glu levels in TMZ-sensitive cells [47]. Glu was also detected in both 2D and 3D cell cultures of established U87 and LN 229 cell lines [38], as well as various self-derived GBM models [35]. Furthermore, researchers have successfully identified Glu in GSC following treatment with a glutaminase inhibitor; as expected glutaminase levels were lower after the administration of the agent [54]. Moreover, an NMR approach has been successfully employed to detect Glu among the intrametabolome of U87 following treatment with TMZ or *Cibotium barometz* polysaccharides [36], and it has also been detected using astrocytoma cell lines derived from glioma tissue [34] and established cell lines [30,55]. The analysis of rat glioma BT4C cells revealed the presence of Glu and lactic acid within the intracellular metabolome, which suggests that these compounds can be used as a target for relatively easy (compared to human trials) investigations with in vivo rat models [44]. Except for α-KG, all of the well-established oncometabolites related with GBM were found within in vitro cell based studies (Table 3) proving the applicability of such approaches for diagnosis purposes, as well as a convenient and easy way for searching for further biomarkers.

## 6. Pharmaco-Metabolomics as a Tool for Glioma Drug Testing In Vitro

Thanks to the extensive work that was conducted to identify potential metabolites for glioma diagnostics and prognostics, cell cultures have emerged as a truly promising model for drug testing and the exploration of tumor resistance to therapy. Knowledge regarding significant pathways and alterations to their metabolism could be used to predict the effectiveness of different therapeutics depending on the phenotype of the cells. For instance, St-Coeur et al. compared TMZ-sensitive and TMZ-resistant U373 cell lines after combined treatment with either TMZ and lomeguatrib (MGMT inhibitor) or TMZ alone and discovered a panel of distinct metabolites that differed among the cell lines. Specifically, they found increased levels of glucose, citrate, and isocitrate in the TMZ-resistant line, and overconcentrations of creatine, PC, Cho and alanine in the TMZ-sensitive GBM cells [47].

Since Gln and glutaminolysis targeting have been previously suggested, Koch et al. examined the influence of glutaminase (GLS) inhibitors on GSC [32,54]. Their pharmaco-metabolomic approach to in vitro studies of the aforementioned inhibitors—in this case, evaluating their effectiveness—allowed for exceptional target specificity. Interestingly, even though both tested inhibitors were found to have a toxic influence on cultured cells, only one of them resulted in actual glutaminolysis suppression [54]. The use of a GLS inhibitor, which can inhibit Glu synthesis in in vitro studies, has also been shown to sensitize gliomas with the IDH1 mutation to oxidative stress by McBrayer et al. [27]. Metabolomics analysis was also successfully used in the study conducted by Shi et al.to evaluate the ability of *Cibotium barometz* polysaccharides (CBPs) to resensitize TMZ-resistant cells. The findings showed changes in the metabolites involved in GSH metabolism (e.g., Glu, Gly, or taurine) and significant accumulation of ROS, thus proving the effectiveness of used compounds [36]. A similar pharmaco-metabolomic approach was used by D’Alessandro et al., to analyze how the Gli1 inhibitor affected murine glioma cells that overexpressed Gli1. This method was able to provide good target specify for the studied drug and its anti-tumor influence, both in vitro and in vivo [31]. This knowledge regarding alterations to the metabolism of Glu, Cho, and Gly in different types of GBM enabled further study of the Notch inhibitor mode of action and the determination of the Notch blockade as a promising target for GBM therapy [35]. In vitro metabolomics have also been successfully used to monitor the potential effect of various drugs on lipid synthesis for compounds such as FK866 inhibitor, phospholipase D (PLD) inhibitor, or gamma-linoleic acid [39,51,53]. It should be noted that some of the studies reviewed earlier also fit within the pharmacometabolomics approach. The full scope of analyzed literature is reported in Table 1. Moreover, metabolomics can also be used to assess cytotoxicity in in vitro applications, for example, particles for gene transfection [58]. The knowledge gained from the basic research discussed in metabolomic paragraph has been successfully used to select markers to determine the efficiency and target specificity of targeted drugs, making metabolomics in vitro an interesting tool for novel targeted therapies development. Gln, Glu, and GSH metabolism being especially useful in determining the effectiveness of analyzed drugs.

## 7. Conclusions

The studies reviewed in this paper highlight the importance of careful test planning for the accurate metabolomic profiling of GBM cells. Factors such as culture model, medium composition, established or patient-derived cell lines, and oxygen levels should all be chosen based on desired aspects of a tumor’s particular microenvironment. Moreover, sample preparation should use only the most effective metabolism quenching or extraction methods. In vitro studies face a problem at the level of in vitro-in vivo extrapolation, as metabolic reactions in a living organism are much more complex than in vitro environments are able to capture. However, with careful design, e.g., the use of 3D culture models, hypoxic conditions when conducting a study, or usage of more efficient sample preparation methods, in vitro studies on GBM metabolomics can be extremely useful for the diagnosis and prognosis of brain tumors, as well as for studying new drugs or mechanisms of drug resistance.

## Figures and Tables

**Table 1:** Metabolites detected in in vitro GBM by metabolomics.

Project Goal	Sample Prep	Instrumental Analysis	Cell Culture Model	Cell Source	Compounds Found	IVIVE	Refrence
Cells differentiation	Intracellular metabolome: PBS wash, MeOH addition, snap freeze in liquid nitrogen, thaw, vortex, centrifugation, supernatant collection, resuspension of cell pellet with water, combining of supernatant and pellet, centrifugation, supernatant transfer and evaporation, reconstitution in 80% MeOH	LC-MS/MS Q-Exactive Orbitrap (Thermo Scientific, Waltham, MA, USA ) ACQUITY UPLC CSH C18 column (2.1 mm x 100 mm, 1.7 mm, Waters); QTRAP 5500 (AB Sciex, Milford, MA, USA) Synergi Hydro-RP column (4.6 mm 250 mm, 4 mm, Phenomenex, Torrance, CA, USA)	2D	U87MG U87MG GSCs	Kynurenie; L-Formylkynurenine; Stearoylcarnitine; Propionylcarnitine; Gamma-Glu–Leu; Acetylcarnitine; Carnitine; Tetradecanoylcarnitine; NAD; LPC (18:0); Pantothenic acid; LPE (18:0); Glutathione; Hypoxanthine Xanthosine; XMP; LPC (15:0); Oxidized glutathione;trans-2-Hexadecenoyl-carnitine; Spermidine; ADP; N-Oleoylethanolamine; LPC (14:0); trans-Cinnamic acid; LPC (20:1); Proline; Valine; 2-Hydroxycinnamic; Leucine; IMP; D-Glucose 6-phosphate; LPC (22:6); Pentanoylcarnitine; Palmitoylcarnitine; Oleoylcarnitine; Guanosine; Methionine sulfoxide; Guanine; Pyrrolidonecarboxylic acid; Creatine; GMP; UMP; N-Acetyl-D-glucosamine; Choline; Tryptophan; Indoleacrylic acid; Glycerophosphocholine; 5′-Methylthioadenosine; Phenylalanine; UDP-N-acetyl-glucosamine; Pantothenic acid; LPE (18:1); UDP-glucose; Tyrosine; N1-Acetylspermine; N1-Acetylspermidine	ND	[42]
Biomarker discovery	Quenching: Ice-cold PBS wash, MeOH add, mechanical scraping chloroform and water add, vortex, orbital shake, centrifugation, transfer of polar phase (methanol:water) into separate vial, evaporation, reconstitution with deuterated water (with 1.5 M KH2PO4 and 0.1% TSP), vortex, centrifugation, supernatant analysis	^1^H NMR Bruker Avance III600 MHz spectrometer, (Billerica, MA, USA)	2D	CHG5 SHG44 U87 U118 U251	Valine; Leucine; Isoleucine; Lysine; Glutamate; Glutamine; Glutathione; Threonine; Tyrosine; Phenylalanine; Taurine; Creatine; Lactate; Glycerophosphocholine; Myo-inositol; Formate; Acetate	ND	[34]
Drug treatment	Extracellular metabolome: cell culture medium collection, centrifugation, store (−80 °C), addition of Na2HPO4:deuterated water and TMSP, pH adjustment with HCL Intracellular metabolome: Cell pellet ice-cold PBS wash ×4, trypsinization, centrifugation, reconstitution with buffer, sonication, centrifugation, freeze, deuterated water with H2O containing 10 mM TMSP add	^1^H NMR Bruker 900-MHz spectrometer, (Billerica, MA, USA)	2D	GL261	Acetate; Acetoacetate; N-acetylaspartate; Alanine; L-alanyl-l-glutamine; arginine; l-asparagine; l-aspartic acid; cadaverine; citrate; creatine; choline; dimethylamine; ethanol; fumarate; formate; d-glucose; glucose-6 phosphate; glutamate; l-glutamine; glycine; l-sistidine; l-isoleucine; lactate; l-leucine; l-lysine; malate; l-methionine; methyloxovalerate; myo-inositol; niacinamide; Puryvate; Succinate; l-phenylalanine; Phosphocreatine; l-threonine; l-tyrosine; l-tryptophan; l-valine;	ND	[31]
Biomarker discovery	Targeted intracellular metabolome: cold PBS wash, cold MeOH:water add, mechanical scraping, transfer into tube, chloroform add, sonication, centrifugation, lyophilization, dissolving with MeOH:water, derivatization with AccQTag kit (Waters, Milford, MA, USA)	Untargeted approach CE-MS Agilent 7100 coupled with 6224 TOF-LC/MS (Agilent Technologies, Santa Clara, CA, USA) Targeted approach Agilent 6460 Triple Quad LC/MS Agilent C18 Column (2.1 mm × 100 mm, 1.8 um (Agilent, Santa Clara, CA, USA)	2D	U251 U87	Cysteine; Hypotaurine; Taurine; Cystine; Cysteinesulfinic acid	Achieved—targeted compounds were found within glioma tissue derived from patients	[43]
Biomarker discovery—ASS negative vs. ASS positive GBM	Extracellular metabolome: Frozen supernatant (−80 °C) thaw, MeOH:water (9:1) add, shake, centrifugation, supernatant transfer evaporation, storage (−80 °C), methoxyamine solution in pyridine add, trimethylsilylation, heptane with methyl stearate add Intracellular metabolome: Frozen cell pellet (−80 °C) thaw, MeOH:water (9:11) add, beads homogenization, centrifugation, supernatant transfer, evaporation, storage (−80 °C), methoxyamine solution in pyridine add, trimethylsilylation, heptane with methyl stearate add	GC-TOFMS Agilent 6980 GC (Agilent, Santa Clara, CA, USA) Pegasus III TOFMS (Leco Corp, St Joseph, MI, USA) DB5-MS Column (10 m x 0.18 mm x 0.18 μm, J&W Scientific, Folsom, CA, USA) 2D GC-TOFMS Pegasus 4D (Leco Corp, St Joseph, MI, USA) coupled with Agilent 6890 GC (Agilent Technologies, Palo Alto, GA, USA) Column BPX-50 (30 m x 0.25 mm x 0.25 μm, SGE) Column VF-1MS (1.5 m x 0.15 mm x 0.15 μm; J&W Scientific Inc, Folsom, CA, USA)	2D	LN229 SNB19 GAMG U118 T98G U87 Normal Human Astrocytes (NHA)	Pyrophosphate; Erythrose-4-Phosphate; Glucaric Acid; 1,4 Lactone; Ribofuranose; Ribose; Ribose-5-Phosphate; Putrescine; Spermidine; Adenine; Hypoxanthine; Uracil; Uridine; Erythritol; Taurine; Tryptophan; Tyrosine; Arginine; Ammonia; Proline; Arginine; Asymetrical-N,N-Dimethylarginine; Citrulline; Ornthine; Citrulline; N-Acetylornithine; Ornithine; 2-Oxoisocaproic Acid; Isoleucine; Leucine; Valine; 1,2-Ethandimine; 1,3,5-Trioxepane; 1-Monostearoylglycerol; 2-Pyrrolidone-5-Carboxylic Acid; Aminomalonic Acid; Cadaverine; Cellotriose; Dihydroxyacetonephosphate; Elaidic Acid; Glucopyranos; N-Acetyl Glutamyl Phosphate; Nonanoic Acid; Phosphoric Acid; Pyrazine; Stearic Acid; Xylitol	ND	[29]
Subtype determination	Intracellular metabolome: Cell harvest by scraping, PBS wash x2, centrifugation, incubation on ice, suspension in ice-cold acetonitrile (50%), incubation on ice, centrifugation, evaporation, dissolve in deuterium oxide	^1^H NMR Bruker Avance III 400 MHz spectrometer, (Billerica, MA, USA)	2D	LN229 VLN319	Taurine; Glutamine; UDP; Glutamate; Choline; Citric acid; Phosphocholine; Aspartate; Glycerophosphocholine; Asparagine; Glycine; Methionine; myo-Inositol	ND	[30]
				HS683 LN405	Valine; Glutamate; Leucine; Citric acid; Isoleucine; Aspartate; Alanine; Asparagine; Lactate; Methionine		
				A172 U343 LN18	GABA; Methionine; Proline; Citric acid; Glutamine; Aspartate; Glutamate; Asparagine;		
				U373 BS149	Succinic acid; Glycerol 3-phosphate; Serine; Glucose; Adenine; cis-Aconitic acid; Taurine; GABA; Lysine; Proline; Tyrosine		
Drug treatment	Intracellular metabolome: Ice-cold PBS wash, cell scraping, centrifugation, cold PBS wash, snap freeze in liquid N2, deuterated water add	^1^ H NMR Varian 600MHz (14.1 T) spectrometer, (Oxford, UK)	2D	BT4C (rat)	Acetate; Alanine; Aspartate; Choline; Creatine; Glutathione; Glutamate; Glutamine; Glycerophosphocholine; Glycine; Lactate; myo-Inositol; PC; Peth; Scyllo-Inositol; Succinate; Taurine; Hypotaurine; Guanosine		[44]
Culture conditions evaluation	Intracellular metabolome: PBS wash, cold MeOH add, cell scrapping, transfer into tube, chloroform add, vortex, water add, vortex, transfer of water:MeOH phase, Chelex-100 add, centrifugation, lyophilization, resolving in deuterated water based buffer with DSS and propionic-2,2,3,3,-d4 acid	1 H NMR Bruker Avance 500 spectrometer, (Billerica, MA, USA) Bruker Avance III HD 600 spectrometer, (Billerica, MA, USA)	2D and 3D	U87	Adenine; myo-inositol; Glycine; PC; Glycerophosphocholine; Free choline; Total choline; Total creatine; Glutathione; Glutamine; Glutamate; N-acetylaspartylglutamate; Alanine; Lactate; Threonine; Valine/isoleucine;	ND	[38]
Biomarker discovery—IDH1 wildtype	Live cells metabolomic: 1-13C-glucose and L-3-13C-glutamine or 2-13C-pyruvic acid add to cell culture medium intracellular metabolome: 1-13C-glucose or 3-13C-glutamine add to cell culture medium, cell trypsinization, centrifugation, cold MeOH addition, vortex, cold chloroform add, cold water add, transfer of MeOH:water phase, lyophilization, reconstitution with deuterated water with TSP	13C-MRS500 MHz INOVA spectrometer (Agilent Technologies, Santa Clara, CA, USA) 1H MRS 13C-MRS spectra 500 MHz Avance spectrometer (Bruker BioSpin, (Billerica, MA, USA) )	2D and 3D	U87 NHA BT54 BT142	Glutamate; 2-Hydroxyglutarate	ND	[45]
Biomarker discovery	Intracellular metabolome: saline wash, cell scraping, transfer into tube, saline wash, centrifugation, cold MeOH:chloroform:water add, centrifugation, resuspension, sonication, centrifugation, supernatant transfer for derivatization and analysis	GC-TOF-MS Agilent 6890 (Waldbronn, Germany), LECO Pegasus 2 TOF (St Joseph, MI, USA)	2D	U87	Citric acid; Cis-aconitic acid; Succinate; Fumarate; Malate; Glucose-6-phosphate; Phosphoenolpyruvic acid; Pyruvate; Lactate; Isoleucine; Leucine; Lysine; Methionine; Phenylalanine; Threonine; Tryptophan; Valine; Cysteine; Tyrosine; Histidine; Alanine; Asparagine; Aspartate; Glutamate; Glutamine; Glycine; Proline; Serine; Ornithine; Hexadecanoic acid; Octadecanoic acid; Octodecenoic acid;Phosphatidyl-l-serine; Ethanolamine; Cholesterol; Glycerol; Glycerol-3-phosphate	ND	[46]
Drug treatment	Cell scraping, PBS wash, centrifugation, pellet PBS wash, centrifugation, resuspension with ACN:water (1:1), ultracentrifugation, supernatant evaporation, dissolving in deuterated water	1H NMR Bruker Avance III 400 MHz spectrometer, (Billerica, MA, USA)	2D	A172, LN18, LN71, LN229, LN319, LN405, U373, U373R*	Phosphorylcholine; Glycerol-3-phosphate; Serine; Choline; Histidine; Succinate; Taurine; Tryptophan; Glycine; Glutathione—reduced; Citric acid; Glutamine; Phosphorylcholine; Leucine; Choline; Lysine; Isoleucine; Alanine; Proline; Glycerol-3-phosphate; Phosphorylcholine; Aconitate; Taurine; Tryptophan; Alanine; Threonine; Valine; Acetone; Aconitate; Adenine; Adenosine; Alanine; Arginine; Asparagine; Choline; Citric; Creatine; Ethanol; Glucose; Glutamate; Glutamine; Glutathione—oxidized; Glutathione—reduced; Glycerol-3-phosphate; Glycerophosphocholine; Glycine; Histidine; Isocitrate; Isoleucine; Lactate; Leucine; Lysine; Methionine; myo-Inositol; Oxoglutarate; Phenylalanine; Phosphorylcholine; Proline; Serine; Succinic Acid; Taurine; Threonine; Tryptophan; Valine	Most compounds were found in primary GBM tissue	[47]
Drug treatment	Scrapping with cold PBS in deutered water, 2x wash, filling 50 µL inserts with cells, snap-freezing	HR-MAS Bruker 500 MHz spectrometer, (Billerica, MA, USA)	2D	U87	myo-Inositol; Glycerophosphocholine; Lipids; CH = CH; CH = CHCH2CH = CH	ND	[48]
Drug treatment	Intracellular metabolome: PBS wash, cold MeOH:water (4:1), ultracentrifugation, transfer into vial	LC-MS Agilent 1290, Agilent 6520 TOF (Santa Clara, CA, USA) column: Waters Acquity UPLC BEH (bridged ethyl hybrid) Amide 1.7 μm 2.1 × 100 mm HILIC, (Milford, MA, USA)	2D	Res259 Res186 BT66 JHH-NF1-PA1	Glutamine; Glutamate; Glutathione	Achieved—similar pathways were found in vivo in patient derived xenograft in mice	[49]
Drug treatment	Intracellular phosphometabolome: Cold saline wash, trypsinization, centrifugation, perchloric acid add, sonication, neutralization with KOH, ultracentrifugation, Chelex-100 add, filtration, pH adjustment, lyophilization, dissolving in deuterated water Intracellular phospholipidome: Cold saline wash, cell scrapping, transfer to tube prefilled with cold MeOH, chloroform add, shake, separation funnel filter, KCL wash, overnight separation, chloroform phase collection, evaporation, dissolving in chloroform, MeOH:EDTA add	31P MRS Varian Inova500, (Oxford, UK)	2D, 3D and cocultures	C6	Phosphatidic acid; Cardiolipin; Plasmenyl phosphatidylethanolamine; phosphatidylethanolamine; Phosphatidylserine; Sphingomyelin; Phosphotidylinosine; Plasmenyl phosphatidylcholine; Phosphatidylcholine	ND	[50]
Drug treatment	cell centrifugation, pellet resuspension in water, MeOH:chloroform with BHT add, periodical vortex, chloroform and KCL add, vortex, centrifugation, chloroform phase collection, evaporation, reconstitution in MeOH:chloroform (1:1)	LTQ-Orbitrap Elite instrument 538 (Thermo Fisher Scientific, Waltham, MA, USA) equipped with a robotic 539 nanoflow ion source TriVersa NanoMate (Advion BioSciences, Ithaca, NY, USA quantification with GC-MS GCMS-QP2010, Shimadzu, (Japan), column: 10 m × 0.1 mm ID, 0.2 μm film thickness	2D	U87	Cholesteryl ester; Cardiolipin; Glucosylceramide; Lysophosphatidylcholine; Lysophosphatidylethanolamine; Phosphatidic acid; Phosphatidylcholine (diacyl); Phosphatidylcholine (alkyl–acyl); Phosphatidylethanolamine (diacyl); Phosphatidylethanolamine plasmalogen (alkenyl–acyl); Phosphatidylglycerol; Phosphatidylinositol; Phosphatidylserine; Sphingomyelin; Triacylglycerol	ND	[51]
Biomarker discovery	Intracellular metabolome: Cell dissociation, PBS wash, centrifugation, freeze, upon analysis deuterated water add	NMR Bruker Avance III spectrometer (Bruker BioSpin, Billerica, MA, USA)	2D, 3D and mixed 2D/3D	Primary glioblastoma	Acetate; Alanine; Choline; Creatine; GABA; beta-Glucose; Glutamate; Glutamine; Glycerophosphocholine; Glycine; lactate; myo-Inositol; N-Acetylaspartate; PC; Serine; Taurine; Valine	Achieved—some pathways altered in 3D and 2D/3D matched pathways in patient tumor relapse	[52]
Drug treatment	Intracellular metabolome: PBS wash, cold MeOH add, cell scraping, transfer into tube, chloroform add, vortex, water add, vortex, separation of water:MeOH phase, Chelex-100 add, centrifugation, lyophilization, resolving in deuterated water with TSP	1H NMR Bruker Avance 500 spectrometer, (Billerica, MA, USA)	3D	Self-derived cell lines: GBM1 040922 GBM1016 GBM1417 commercial cell lines: LN229, U87	Valine/Isoleucine; Threonine; Lactate; Alanine N-acetylaspartylglutamate; Glutamate; Glutamine; Glutathione; Total Creatine; Free Choline; PC; Glycerophosphocholine; Glycine; myo-Inositol	ND	[35]
Drug treatment	Targeted intracellular metabolome: ice-cold PBS add, cell scraping centrifugation, pellet resuspension with MeOH:water (7:3), agitation, incubation in −20 °C, IS load, agitation, ultracentrifugation, supernatant collection, solvent evaporation, reconstitution with 2 mM ammonium acetate and 3 mM hexylamine solution.	LC-MS/MS MDS SCIEX 4000QTRAP hybrid triple quadrupole/ linear ion trap mass spectrometer (Applied Biosystems, Waltham, MA, USA ) Waters Acquity BEH C18 column (2.1 × 50 mm, 1.7 μ) (Milford, MA, USA)	2D	U87MG	dATP; dCTP; TTP	ND	[53]
		Acquity HSS T3 column (2.1 Å~ 100 mm, 1.8 μm).			Carbamoyl aspartate; Orotic acid		
Biomarker discovery	PBS wash, centrifugation, pellet resuspension with deuterated water and TMSP, centrifugation	1H NMR Advance spectrometer (Bruker, AG, Darmstadt, Germany)		T98G primary glioma cells and neural stem/progenitor cells	myo-Inositol; UDP-hex; N-Acetylaspartate; O-2A; Glycine; Aspartate; O-2A; Total Creatine; Glycine; Lip; Glutamine; GSH; Glutamate; GABA; GalNAc;	ND	[37]
Therapeutic targets/drug treatement	Intracellular metabolome: Cell harvest, PBS wash, ice-cold NaCL (0.9 mM) wash x2, suspension in ice-cold H2O, of ice-cold MeOH add, vortex, incubation, ice-cold chloroform add, vortex, incubation, ice-cold H2O add, vortex, incubation, centrifugation, water-methanol phase collection, Chelex-100 add, filtration, evaporation, freezing (−80 °C) lyophilization	1H NMR Bruker AVANCE III HD 700 spectrometer 700 MHz (Billerica, MA, USA))	2D and 3D Tissue samples	JHH520 GBM1 23, 233, 268, 349 407 SF188 NCH644	Alanine; Aspartate; Glutamine; Glutamate; Glycine; Glutathione; Lactate; Myo-inositol; PC; Succinate; Tricarboxylic acid; Total choline; Total creatin	ND	[54]
Therapeutic targets assesement	Intracellular metabolome GC-MS HOG, NHA: ice-cold saline wash, culture plate snap freeze with liquid N2, cold MeOH:water (7:3) add, chloroform add, vortex, centrifugation, MeOH:water phase separation, evaporation GSC lines: cold saline addition, neurosphere transfer into tube, centrifugation, freeze of pellet with liquid N2, cold MeOH:water (7:3) add, chloroform add, vortex, centrifugation, MeOH:water phase separation, evaporation Derivatization with methoxyamine and N-(tert-butyldimethylsilyl)-N-methyl-trifluoroacetamide/1% tert-butyldimethylchlorosilane LC-MS/MS performed as for GC-MS with exception that MeOH:water (4:1) was used and dried extract were resuspended with water Extracellular metabolome medium collection, MeOH, Water (7:3) add, rest as above for GC-MS and LC-MS/MS	GC-MS using an Agilent 7890A (Santa Clara, CA, USA) 5500 QTRAP hybrid triple quadrupole mass spectrometer (AB/SCIEX, Framingham, MA, USA), Amide HILIC chromatography (Waters, Milford, MA, USA)		NHA HT1080 HOG IDH1 R132H mutant IDH2 R172K mutant HCT116 NCI-H82 HEK293T GSC lines: TS603, TS516, TS676, MGG152 BT054 BT260	Glutamate; 2-Hydroxyglutarate; alpha-Ketoisocaproate; Valine; Leucine; Isoleucine; alpha-Keto-beta-methylvalerate	Achieved—increased BCAT activity in vitro and in vivo in xenograft mice	[27]
Drug treatment	Intracellular metabolomie: 3× freeze/thaw cycles of water based cell suspension, cold MeOH add, agitation, chloroform add, agitation, ultracentrifugation, collection of chloroform phase, evaporation, reconstitution with TMS:deuterated MeOH Intracellular lipidomice: Cold PBS wash, cell scraping on dry ice, freeze, sonication, centrifugation, pellets resuspend in water, centrifugation, pellet snap freeze on dry ice, storage (−80 °C), extraction: resuspension in water, probe sonication, bath sonication, MeOH:water spiked with IS add, vortex, ice bath incubation, cold chloroform add, incubation 1 h, ultracentrifugation, separation of MeOH;water and chloroform phases, ACN:W (1:1) add, centrifugation, evaporation, snap freezing with dry ice, −80°C storage, combining of both phases in MeOH:ACN:water buffer live cell culture imaging	1H NMR Bruker Avance III 600 MHz spectrometer (Structural Biophysics Laboratory, NCI, Frederick, MD, USA) LC-TOF Q-TOF SYNAPT G2 Si (Waters Corporation, (Milford, MA, USA) Acquity UPLC CSH 1.7 m, 2.1 x 100 mm column (Waters Corp., Milford, MA, USA Raman spectroscopy DXR2xi Raman microscope (ThermoFisher Scientific, Madison, WI, USA)	2D	HT1080	Lipidomics: 1-O-eicosanoyl-Cer d18-1,16-0; 1-O-tricosanoyl-Cer d18-1,18-0; 5-methyldeoxycytidine; Acetylcysteine; Cholesteryl Ester—CE 31-0; Cer d45-1; Cer d50-2; Cer d51-1; PhytoCer t48-1; PhytoCer t53-1; Diacylglycerol: 46-5, 56-9, 57-0, 60-0, 61-1, 64-0, 64-1, 66-1, 67-0, P-36-3, P-39-0, P-43-0, P-44-4, P-48-0, P-48-4, P-49-0, P-50-0, 60-0, P-51-0 Dopamine; Dopamine quinone; pinephrine sulfate; GluCer d39-0; Glutaminyl-arginine; Glutaminylcysteine; Glyceraldehyde; Isovaleric acid amine; Isovalerylglutamic acid; LacPhytoCer t50-0; L-histidine; Methyldeoxycytidine; N2,N2-dimethylguanosine; N-acetyldopamine; N-succinyl-2-amino-6-ketopimelate;O-tricosanoyl-N-hexadecanoyl PA: 43-2, 49-4, 52-4, O-41-0; PC: 22-4, 21-0, 39-6, 40-3; PE: 40-2, 49-4; Phosphoglycolic acid; PI P-36-4; PS 43-2; Pyroglutamic acid; Pyrrolidonecarboxylic acid; Sn-glycero-3-phosphoethanolamine; S-Succinyldihydrolipoamide; Succinyl acetoacetate; TG 15-0,18-1,14-1	Achieved—decrease in lipids observed via Raman imaging microscopy both in vitro and in vivo after dug treatement	[39]
Biomarker discovery	Intracellular metabolome: Cell harvest, PBS wash, centrifugation, incubation on-ice, cold acetonitrile:water (1:1) resuspension, centrifugation, freeze drying, D_2_O add Extracellular metabolome: Medium supernatant filtration, storage (−80 °C), mixing with D_2_O Exosomal metabolome: ultracentrifugation, PBS wash, centrifugation, incubation on-ice, cold acetonitrile:water (1:1) resuspension, centrifugation, freeze drying, D_2_O add	1H NMR Bruker 600 MHz spectrometer, (Billerica, MA, USA)	2D	U118 LN-18 A172 NHA	Formate; Asparagine; Taurocholic acid; Glycerol; Malate; Niacinamide; Lactate; Acetone; 5-oxoproline; Citrate; Proline; Succinate; Ethanol; GSH; GABA; G6P; Isoleucine; Glucose; Taurocholate; Homoserine; Glycine; Carnitine; GSSG	ND	[55]
Drug treatment	culture plates place on ice, cold PBS wash, cell scraping into PBS, transfer into tube, cold MeOH add, sonication, centrifugation, supernatant transfer evaporation, reconstitution in deuterated water with TMSP	1H–NMR AVANCE III 600M NMR Bruker (Germany)	2D	U87	Leucine; Alanine; Creatine; Glutamate; Glycine; Lactate; myo-Inositol; Glycerophosphocholine; Isoleucine; Taurine; Glutathione; Lysine; NAD+; UDP–NAG	ND	[36]
Biomarker discovery/Culture conditions evaluation	intracellular metabolome: cold MeOH add, water add, grinder homogenization, sonication, ultracentrifugation, lyophilization, resuspension with deuterated water	1H-NMR Bruker Avance III HDX 600-MHz FT-NMR Spectrometer, Billerica, MA, USA)		primary	Alpha-ketoglutarate; Succinic acid; Glutathione; Fumarate; Dodecanoic acid; Caproic acid; N-Acetylserotonin; Stachyose; Glyceraldehyde; Serine; Fructose; Lysine; Arginine; Glucose-6-phosphate, Selenomethionine; Glycine; Choline; Guanidoacetic acid; Guaiacol; Oxoglutaric acid; Gamma-Aminobutyric acid	Achieved—similar spatial differences of the metabolic environment	[56]
drug treatment	extracellular amino acid profiling: medium transfer, sulfosalicylic acid add, buffer add, labeling with aTRAQ™(Sciex, Milford, MA, USA), incubation, evaporation, resuspension	C-MS/MS C18 Column Reverse Phase (5 µm, 4.6 mm × 150 mm)	2D	primary U87-MG	Serine; Methionine; Glycine; Tyrosine; Aspartic acid; Isoleucine; Alanine; Leucine; Threonine; Norleucine; Glutamate; Phenylalanine; Histidine; Proline; Arginine; Methionine sulfoxide; Cystine; Lysine; Valine; Norvaline	ND	[32]
Biomarker discovery/Culture conditions evaluation	extracellular metabolome: medium collection, ACN add, −80 °C store untill analysis, dilution intracellular metabolome: cold PBS wash, cold ACN add, −80 °C short incubation (3 min), cell scrapping, transfer into tube, cold water add, freeze/thaw lysis with vortex (3× times), ultracentrifugation, supernatant store at −80 °C	LC-QTOF 6520 Accurate Mass Q-TOF LC/MS (Agilent Technologies, Santa Clara, CA, USA) biomarker validation 6430 Triple Quad LC/MS (Agilent Technologies, Santa Clara, CA, USA), doczytac czy do metabo byl tez ten MS reverse-phase C18 stable bond column (2.1 mm × 50 mm × 1.8 µ) (Agilent Technologies, Santa Clara, CA, USA).		primary U118 U87 LN18 LN229 NHA	Kynurenine; Tryptophan; Methionine; 5′-methylthioadenosine; S-adenosylmethionine; S-adenosylhomocysteine	Achieved—methionine was found in ex vivo in fresh glioblastoma biopsy tissue	[28]
Therapeutic targets assessment	Intracellular metabolome: Ice-cold PBS wash, cell lysis with dry ice/methanol − 80°C, (80% methanol), scrapping, centrifugation, supernatant collection	UHPLC/MS Waters Acquity UHPLC (Waters Corporation, Milford, MA, USA) LTQ mass spectrometer (Thermo Fisher Scientific Inc. Madison, WI, USA) GC/MS Thermo-Finnigan Trace DSQ MS (Thermo Fisher Scientific, Inc. Madison, WI, USA)	2D in hypoxia	U87	Aldolase; Enolase 2; Glucose-6-phosphate isomerase; Hexokinase; Lactate dehydrogenase A; Pyruvate dehydrogenase kinase 3; Phosphofructokinase; Phosphofructo-2-kinase/fructose-2,6-biphosphatase; Phosphoglycerate mutase; Phosphoglycerate kinase 1; Pyruvate kinase isoenzyme type-M2	ND	[57]
Gln deprivation influence	Intracellular metabolome: Ice-cold PBS wash x3, H2O:MeOH:acetonitrile (2:5:3) add, centrifugation, supernatant collection Extracellular metabolome: Culture media dilution with H2O:MeOH:acetonitrile (2:5:3), centrifugation, supernatant collection	HPLC-MS ZIC-HILIC (SeQuant) with a guard column (Hichrom) Exactive Orbitrap mass spectrometer (Thermo Scientific, Madison, WI, USA)	2D	MOG-G-VW LN-18 LN-229 SF-188 U-251 MG U-87 MG Primary rat astrocytes Primary human GBM: E2 R10 R24	Glutamine; Leucine; Isoleucine; Serine; Valine; Alanine; Lysine; Cysteine S-S; Threonine; Arginine; Proline; Methionine; Asparagine; Ornithine; Taurine; Phenylalanine; Tyrosine; Citrulline; Histidine; Tryptophan; Aspartate; Glycine; Glutamate; Pyruvate; Lacate	ND	[26]
Nanoparticles toxicity	intracellular metabolome: ACN:MeOH (1:1) with α-cyano-4-hydroxycinnamic add onto cells	MALDI-MS/MS MALDI LTQ-XL instrument (Thermo Scientific, Madison, WI, USA)	2D	NG97	2-hydroxy-eicosanoic acid; Docosapentaenoic acid/octadecanoic acid (stearic acid); N-oleyl-alanine; N-stearoyl-alanine;	ND	[58]
Stem-like cells metabolome evaluation	Intracellular metabolome 2D culture: Cold ammonium acetate wash, snap-freezing in liquid nitrogen, ice-cold MeOH:H2O (4:1) add, scrapping, mix, centrifugation, supernatant collection Intracellular metabolome 3D culture: Neurospheres collection, cold ammonium acetate wash, snap-freezing in liquid nitrogen, ice-cold MeOH:H2O (4:1) add, mix, centrifugation, supernatant collection	LC-MS DIONEXUltimate 3000 UPLC HILIC column (AcclaimMixed-Mode HILIC-1, 3 μm, 2.1 × 150 mm) Q Exactive mass spectrometer (QE-LC-MS(Thermo Scientific, Madison, WI, USA)	2D and 3D	U87 NCH644—patient derived stem-like cells	Carbomyloaspartate; Citruline; Proline; Arginine; Aspartate; Ornithine	ND	[59]
IDH1-mutant glioma metabolic reprogramming	intracellular metabolome: cell trypsinization, centrifugation, cold MeOH add, vortex, cold chloroform add, cold water add, separation of MeOH:water phase, lyophilization, reconstitution with deuterated water with TSP	1H--MRS 600 MHz Bruker Avance spectrometer (Bruker Biospin, Rheinstetten, Germany)	2D	U87 NHA with or without IDH1 mutation	Aspartate; Glutamate; Glutamine; Glutathione; Lactate; myo-Inositol; PC; Glycerophosphocholine; 2-Hydroxyglutarate; alfa-Butyrate; Creatine; Hydroxybutyrate; Valine	ND	[33]

**Table 2:** Sample preparation techniques used for in vitro GBM cell lines.

Sample Prep Technique	Instrumentation	Simplicity (Number of Steps)	Derivatization Step Included	Advantages	Disadvantages	Reference
dual-phase extraction	1H NMR	complicated (10–19)	-	broad metabolome coverage: polar metabolites and lipids	time consuming, phase separation required, lyophilisation: additional lab equipment needed	[32,33,34,37,38,44,53]
	1H MRS					
	LC-MS					[26,38,50]
	GC-MS		+			[26]
liqiud-liquid extraction	1H NMR	easy (1–11)	-	no sample prep required	low sensitivity	[29,30,35,36,43,46,47,51,54,55]
	LC-MS		+	quantification included	Can be consider, dirty’ for instrumentation: frequent maintenance needed	[31,42]
			-	High sensitivity, broad metabolome coverage		[25,27,41,48,52,56,58]
	MALDI-MS		-	fast	low metabolite coverage	[57]
	GC-MS		+	High sensitivity, broad metabolome coverage	bead homogenization requires additional lab equipment [28]	[28,45]
	31P MRS	complicated (12)	-	broader metabolome coverage: phosphometabolites and phospholipids	lyophilisation: additional lab equipment needed	[49]
none (live imaging)	13C-MRS	easy (1)	-	live imaging, possibility of time-course cell culture monitoring	targeted approach, low metabolite coverage	[44]
	Raman spectroscopy	easy (0)	-	possible application to tissue analysis suitable for imaging	direct annotation of individual compounds not possible	[38]
liquid-liquid extraction	31P MRS	complicated (12)	-	broader metabolome coverage: phosphometabolites and phospholipids	lyophilisation: additional lab equipment needed	[49]

**Table 3:** In vivo-in vitro extrapolation of oncometabolites in the reviewed literature.

Compound	In Vitro Model	In Vivo/Ex Vivo Investigation
**NAA**	Primary glioblastoma [52] T98G and primary [37]	[87,88,89,90]
**2HG**	U87, NHA [45] U87, NHA, BT54, BT142 [45]	[88,89,90,91,92]
**Glu**	U87 [46] A172, LN18, LN71, LN229, LN319, LN405, U373, U373R [47] Res 259, Res186, BT66, JHH-NF1-PA1 [49] Primary glioblastoma [52] U 87 [38] Self-derived cell lines: GBM1, 040922, GBM1016, GBM1417; commercial cell lines: LN229, U87 [35] T98G and primary [37] JHH520 GBM1, 23, 233, 268, 349, 407, SF188, NCH644 [54] U87 [36] Primary, U87-MG [32] MOG-G-VW, LN-18, LN-229, SF-188, U-251 MG, U-87 MG, Primary rat astrocytes, Primary human GBM: E2, R10, R24 [26] U87, NHA [33] U87, NHA, BT54, BT142 [45] CHG5, SHG44, U87, U118, U251 [34] LN229, VLN319m [30] BT4C (rat) [44]	[87,92,93,94]
**α-KG**	Not found	[93,94]
**PC**	BT4C (rat) [44] Primary glioblastoma [52] U87 [38] Self-derived cell lines: GBM1, 040922, GBM1016, GBM1417; commercial cell lines: LN229, U87 [35] HT1080 [39]	[87,95,96,97,98]
**Lactic acid**	LN229, VLN319 [30] U118 LN-18 A172 NHA [55] U87 [46] A172, LN18, LN71, LN229, LN319, LN405, U373, U373R [47] Self-derived cell lines: GBM1, 040922, GBM1016, GBM1417; commercial cell lines: LN229, U87 [35] U87 [36] CHG5, SHG44, U87, U118, U251 [34] BT4C (rat) [44]	[88]
**Palmitic acid**	U87 [46]	[88,99,100]
**Stearic acid**	NG97 [58] LN229, SNB19, GAMG, U118, T98G, U87, NHA [58]	[88,100]

## Data Availability

Not applicable.
